# Welcome to *One Health Outlook*

**DOI:** 10.1186/s42522-019-0005-y

**Published:** 2019-11-27

**Authors:** Albert Osterhaus

**Affiliations:** Research Center for Emerging Infections and Zoonoses, University of Veterinary Medicine Hannover Foundation, Hannover, Germany

Welcome to the first issue of **‘One Health Outlook’**, the official journal of the One Health Platform.

The **One Health Platform** is a Scientific Reference Network that aims to enhance understanding of and preparedness for current and future outbreaks of zoonoses, emerging and re-emerging infectious diseases in humans and animals as well as a globally increasing problem of antimicrobial resistance. This understanding should be based on the recognition that human, animal and ecosystem health are inextricably linked, and can have profound effects on human society.

Until the start of the last century, infectious diseases caused about 50 % of fatal human diseases worldwide. In the industrialized world, this decreased to less than a few percent in the following decades, largely due to the implementation of public health measures, and the development of vaccines and antimicrobial drugs. Major successes were the eradication of smallpox and rinderpest through well-orchestrated vaccination campaigns in humans and cattle, respectively. Such successes prompted policymakers and scientists to speculate that infectious diseases of humankind and of their domestic animals would eventually be brought under control, at least in the industrialized world. Paradoxically the following decades confronted the world with an ever-increasing number of emerging infectious diseases, some causing true human or animal pandemics. Pathogens spilling over from wildlife reservoirs, either directly or via intermediate hosts, caused most of these disease outbreaks. AIDS from chimpanzees, avian flu from migratory birds, SARS, MERS, and Ebola from bat reservoirs, and arbo-virus infections like Zika transmitted by mosquitos or ticks are striking examples.

In the same period also the globally increasing problem of antimicrobial resistance emerged. A complex mix of predisposing factors linked to major changes in our societal environment and global ecology, collectively created opportunities for pathogens to infect and adapt to new animal and/or human hosts and develop resistance to antimicrobial drugs. This paved the way for the unprecedented spread of infections in humans and animals with dramatic consequences for public and animal health, animal welfare, food supply, economies, and biodiversity. Due to the complex and interactive nature of these predisposing factors, it is virtually impossible to predict which pathogen will strike when in the future. However, a better understanding of the underlying processes may help to develop measures to improve our preparedness for disease outbreaks in humans and animals.

It became clear that there is an urgent need for countries to have the capability and capacity to maintain effective early alert and response systems to detect and quickly react to outbreaks of international concern, and to share information about such outbreaks rapidly and transparently. Responding to such epidemic and pandemic threats requires global cooperation and global participation. Combined with the growing globalization of biological health risks and recognizing the importance of the human-animal-ecosystem interface in the evolution and emergence of pathogens, a One Health approach to tackle these problems seems to be the only rational solution.

The problem of emerging infections is largely paralleled by medical, veterinary, technological, and scientific progress, continuously spurred by our never-ending combat against emerging pathogens. Investment in the better understanding of the human-animal-environmental interface will offer us a head start in the never-ending battle against infectious diseases. Making this knowledge accessible to policy makers to combat these problems at the source, is one of the priorities of the One Health Platform.

As a Scientific Reference Network, the One Health Platform has united some of the best One Health researchers and global experts in its Scientific Advisory Board (SAB), several of whom are members of the **Editorial Board** of the One Health Outlook journal. This Editorial Board consists of leading experts with largely interdisciplinary research performances in all relevant fields of established and emerging infectious diseases, pathology, immunology, epidemiology, medical and veterinary sciences and public health.

The ‘One Health Outlook’ journal -as the official journal of the One Health Platform - aims to become a high impact journal that provides the stage for rapid communication of high-quality scientific knowledge in the field of One Health Science, including antimicrobial resistance, acknowledging the importance of the complex mix of underlying ecological and environmental drivers that facilitate the emergence of these problems.

The ‘One Health Outlook’ journal will give special attention to contributions related to pathogen discovery, determinants governing transmissibility and transmission of established and newly emerging pathogens within and between species, their pathogenesis and intervention strategies as part of biological threat reduction and biosecurity. In the field of antimicrobial resistance special attention will be given to prevalence, surveillance and epidemiology of resistance development in humans, animals, the food chain and the environment, while addressing classical and novel innovative mitigation and intervention strategies. Contributions addressing societal concerns with a One Health component, like the effects of climate change on the emergence and spread of vector-borne pathogens, food safety and the economic impact of these biological threats also will be encouraged.

At regular intervals the One Health Outlook Journal will publish invited review articles on subjects that are of major importance to the One Health Science community.

The Editors have chosen for the format of an open access online-only journal by **Springer Nature** which will allow - as much as possible - the global One Health Science community, affordable and timely access to its content.

